# Springback Behavior and Stroke-Based Compensation Strategy for Roll-Based Bending of High-Strength Aluminum Extrusions

**DOI:** 10.3390/ma19153275

**Published:** 2026-08-03

**Authors:** Jeongsik Lim, Hyungjun Kim, Jinho Kim, Hoyoung Kang, Changhwan Seo, Haeyong Yun

**Affiliations:** 1 Gyeongbuk Technopark, 120 Gongdan 7-ro, Jillyang-eup, Gyeongsan 38463, Republic of Korea; jslim@gbtp.or.kr (J.L.); hykang@gbtp.or.kr (H.K.); 2 Department of Mechanical Engineering, Yeungnam University, Gyeongsan 38541, Republic of Korea; happykim96@yu.ac.kr; 3 R&D Center, ALUS Co., Ltd., 76 Yeongudanji-ro, Ochang-eup, Cheongju 28116, Republic of Korea; 4 Korea Institute of Robotics & Technology Convergence, 1486-20 Gyeongdong-ro, Andong 36728, Republic of Korea; hyyun@kiro.re.kr

**Keywords:** high-strength aluminum extrusion, roll-based bending, springback behavior, feeding displacement, stroke-based compensation

## Abstract

High-strength aluminum tubular extrusions are being increasingly used in lightweight automotive structures; however, the significant springback caused by elastic recovery after bending compromises geometric accuracy. In roll-based bending processes, springback behavior is affected by multiple process parameters, which necessitates a systematic investigation and a simulation-based springback compensation approach. In this study, the springback behavior of high-strength aluminum tubular extrusions subjected to roll-based bending was investigated via finite-element analysis. The effects of feeding displacement, roller rotational speed, and internal mandrel application on springback were comparatively evaluated. Springback was quantitatively assessed based on the displacement and angular deviation after unloading, and the influence of each process parameter was analyzed. Based on the results, variations in roller rotational speed had a negligible effect on springback, whereas feeding displacement was identified as the dominant governing factor under all investigated conditions. Although internal mandrel application reduced stress levels and mitigated wrinkle formation, the overall springback tendency remained dependent on feeding displacement. Based on these findings, a simulation-based stroke compensation approach was proposed, and a compensation window satisfying the target curvature radius of R = 150 mm was numerically identified under the investigated finite-element conditions.

## 1. Introduction

The demand for lightweight structures in the automotive and mobility industries continues to increase in response to the need for improved fuel efficiency and reduced carbon emissions. Accordingly, the use of aluminum alloys in structural components has substantially increased. In particular, aluminum extrusions have been widely adopted for automotive structural units, such as body reinforcements, side members, and battery-pack frames, owing to their high specific strength, excellent corrosion resistance, and flexibility in cross-sectional design [1,2]. These advantages allow aluminum extrusions to satisfy both structural stiffness and crashworthiness requirements. Nevertheless, aluminum extrusions are prone to significant springback after forming, which makes it difficult to achieve the desired geometric accuracy, especially when high-strength alloys are used. Springback is a representative forming defect caused by elastic recovery after plastic deformation and is influenced by material properties, deformation history, and forming conditions. In bending processes, springback behavior is strongly associated with the bending moment distribution and asymmetric deformation within the cross-section [3,4]. Consequently, springback has been recognized as one of the most critical challenges in forming processes involving aluminum and high-strength alloys, and extensive efforts have been devoted to predicting and controlling it.

Previous studies on springback have mainly focused on press-based bending processes, in which springback compensation is commonly achieved through die-shape modification or iterative finite-element simulations [5,6,7]. Although these approaches have proven effective for sheet-metal forming, their direct applicability to aluminum tubular extrusions is limited because of the complex cross-sectional geometries and continuous contact conditions involved in extrusion bending. Moreover, iterative compensation strategies often require high computational cost and are not always practical for industrial process design [8,9]. In contrast, roll-based bending processes involve continuous roller–material contact and feeding-driven deformation mechanisms, which fundamentally differ from those of conventional press-based bending. Previous studies have reported that contact length, feeding conditions, and roller geometry play critical roles in determining deformation behavior and residual stress distribution during the roll-based bending of profiles [10,11,12]. In particular, feeding conditions directly affect the contact state between the rollers and the extrusion, thereby influencing the bending moment distribution and the resulting springback behavior [13,14]. Recent studies have further emphasized the importance of process-parameter control and numerical compensation in the bending and forming of aluminum profiles and high-strength aluminum alloys [13,14,15,16]. In particular, finite-element-based approaches have been increasingly used to evaluate springback tendency, residual stress evolution, and compensation strategies under complex forming conditions. Nevertheless, most previous studies have focused on either sheet-metal springback or general profile bending behavior, whereas systematic investigations of stroke-based compensation in roll-based bending of high-strength aluminum tubular extrusions remain limited.

Despite these previous efforts, the relative effects of key process parameters, such as feeding displacement, roller rotational speed, and internal support conditions, on the springback behavior of high-strength aluminum tubular extrusions in roll-based bending have not yet been systematically and quantitatively evaluated. Furthermore, although many studies have focused on identifying springback trends, practical compensation strategies that can be readily implemented in process design remain limited [17,18,19,20]. This limitation becomes even more pronounced for high-strength aluminum alloys. In particular, 7xxx-series aluminum alloys have attracted considerable attention for structural reinforcement components because of their superior strength-to-weight ratio. However, their high strength and elastic recovery characteristics often lead to pronounced springback and reduced formability [5,21,22,23]. Therefore, a comprehensive understanding of springback behavior and an effective compensation strategy tailored to roll-based bending processes are essential for facilitating the industrial application of high-strength aluminum tubular extrusions.

In this study, the springback behavior of high-strength aluminum tubular extrusions subjected to roll-based bending was investigated through finite-element analysis. The effects of feeding displacement, roller rotational speed, and internal mandrel application on springback were quantitatively evaluated to identify the dominant governing parameters. Furthermore, an effective stroke-based springback compensation strategy based on changes in tool–material contact geometry was proposed, and a compensation window satisfying the target curvature radius of R = 150 mm was determined. The findings of this study provide practical guidelines for improving geometric accuracy in roll-based bending processes for high-strength aluminum tubular extrusions.

## 2. Numerical Modeling and Springback Evaluation

### 2.1. Finite-Element Model for Roll-Based Bending

**Figure 1:**
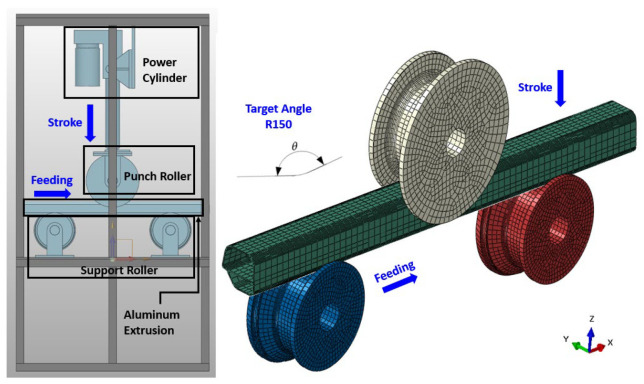
Schematic of roll-based bending process and finite-element model.

A 3D finite-element analysis was performed to investigate the springback behavior of high-strength aluminum extrusions subjected to roll-based bending. Roll-based bending processes are characterized by continuous roller–material contact and feeding-driven deformation mechanisms, which have been reported to significantly influence deformation history and residual stress distribution [10,11]. Accordingly, a roll-based three-point bending configuration was adopted to represent the actual forming conditions accurately. Accordingly, a roll-based three-point bending configuration was adopted to represent the actual forming conditions accurately, as shown in Figure 1.

The finite-element model consisted of an upper punch roller, lower support rollers, and an aluminum extrusion. Bending deformation was triggered by the vertical displacement of the punch roller, while the extrusion was fed through the rotational motion of the support rollers. All rollers were modeled as rigid bodies, whereas the aluminum extrusion was modeled as a deformable body to capture both its bending deformation and subsequent springback behavior. The contact between the rollers and the extrusion was defined using a surface-to-surface contact formulation, since contact conditions critically influence the deformation behavior and springback response in roll-based bending processes [13]. The detailed material properties and contact parameters are described in Section 2.2.

The detailed geometric dimensions of the commercial aluminum extrusion profile and roll-bending apparatus cannot be fully disclosed due to confidentiality restrictions related to the industrial partner. Therefore, the present study focuses on the relative comparison of springback behavior under different process parameters rather than the complete reproduction of a specific commercial forming system. The same finite-element modeling strategy, material model, contact definition, and boundary conditions were consistently applied to all simulation cases to ensure a reliable comparison of the effects of feeding displacement, roller rotational speed, and internal mandrel application.

### 2.2. Material Properties and Contact Conditions

**Figure 2:**
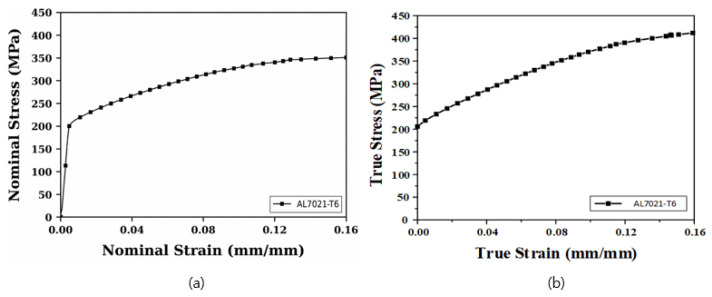
Stress–strain curves of AL7021-T6 used for material characterization: (**a**) nominal stress–strain curve obtained from the uniaxial tensile test and (**b**) converted true stress–true strain curve used as the input flow stress data for the finite-element simulation.

The material behavior of the aluminum extrusion was characterized based on experimentally obtained uniaxial tensile test data. The nominal stress–strain curve obtained from the tensile test is shown in Figure 2a. The nominal stress–strain data were converted into a true stress–true strain curve, as shown in Figure 2b, and this converted curve was used as the input flow stress data for the finite-element simulation.

An isotropic elastic–plastic constitutive model was employed to represent the nonlinear plastic deformation behavior of the aluminum alloy. The effects of plastic anisotropy and the Bauschinger effect were not explicitly considered in the present model. Therefore, the material model is suitable for comparing relative springback tendencies among the investigated process conditions, although it may limit the absolute accuracy of springback prediction.

The elastic properties and contact parameters used in the simulation are summarized in Table 1. The Young’s modulus and Poisson’s ratio were set to 70,000 MPa and 0.33, respectively. The contact interaction between the rollers and the aluminum extrusion was modeled using a surface-to-surface contact formulation with a Coulomb friction model. The friction coefficient was set to 0.12 and was consistently applied to all simulation cases. Because the actual friction condition in the industrial process may vary depending on lubrication, surface roughness, and contact pressure, the contact condition was defined to support the relative comparison of process-parameter effects rather than the absolute prediction of friction-dependent springback values.

### 2.3. Process Parameters, Target Geometry, and Simulation Conditions

**Figure 3:**
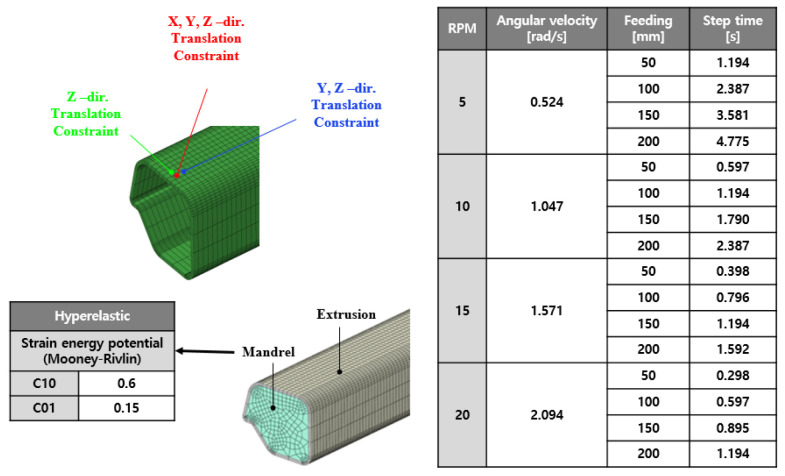
Analysis conditions.

The springback behavior in roll-based bending is influenced by several process parameters, among which feeding displacement, roller rotational speed, and internal support conditions are considered the most relevant. Studies have shown that feeding conditions and contact length dominantly govern the deformation history and residual stress distribution in roll-based bending processes, whereas the influence of roller rotational speed is relatively limited [10,12]. Accordingly, feeding displacement was selected as the primary process parameter in this study, and it was systematically varied to evaluate its effect on springback behavior. Roller rotational speed was treated as an independent variable to assess its influence relative to feeding displacement. Additionally, the effect of internal support conditions was investigated by applying a mandrel in selected simulation cases, which allowed for the influence of internal constraints on deformation stability and springback behavior to be examined. Additionally, the effect of internal support conditions was investigated by applying a mandrel in selected simulation cases, which allowed for the influence of internal constraints on deformation stability and springback behavior to be examined, as shown in Figure 3.

A curvature radius of R = 150 mm was defined as the target geometry for roll-based bending. This radius represents a typical intermediate bending condition frequently required for automotive structural aluminum extrusions, such as reinforcement members and frame components. Moreover, R = 150 mm corresponds to a bending condition that avoids severe instability phenomena, such as wrinkling or fracture, while still allowing springback behavior to be clearly observed. Therefore, this curvature radius was considered an appropriate and representative condition for investigating springback characteristics and evaluating compensation strategies for roll-based bending processes. In all simulation cases, the bending deformation was controlled to achieve the same target curvature of R = 150 mm during the forming stage. Subsequently, an unloading step was performed to evaluate the springback behavior. This simulation procedure enabled a consistent and reliable comparison of the forming responses and springback characteristics under different process conditions.

### 2.4. Definition of Springback Measures

To quantitatively evaluate springback behavior, both displacement-based and angle-based measures were employed. Displacement-based springback was defined based on the geometric deviation of the extrusion centerline after unloading, while the maximum springback displacement was used as an indicator of overall shape recovery. This approach has been reported to be effective for quantifying shape deviation in elongated structural members, such as aluminum extrusions [6,7]. Angle-based springback was defined as the difference between the target bending angle required to achieve the desired curvature and the actual bending angle measured after springback. Angle-based evaluation is particularly useful for process design and compensation strategy development, and it has been widely utilized in springback compensation studies [5,8]. By employing both displacement-based and angle-based measures, springback behavior was comprehensively analyzed under various process conditions. Specifically, angle-based springback was used as the primary metric for developing the proposed stroke-based springback compensation strategy.

### 2.5. Numerical Reliability, Assumptions, and Limitations

To ensure a consistent comparison of the numerical results, the same modeling strategy, material model, contact formulation, boundary conditions, mesh strategy, and unloading procedure were employed across all simulation cases. This approach is appropriate for the objective of this study, which is to compare the relative springback behavior under different process parameters rather than to predict the absolute springback response of a specific commercial forming system.

A separate experimental validation, mesh convergence study, or time-step sensitivity analysis was not included in the present work. Therefore, the numerical values reported in this study should not be overinterpreted as exact absolute predictions. Instead, the results are used to identify relative tendencies associated with feeding displacement, roller rotational speed, and internal mandrel application. The limitations associated with the isotropic material model, friction modeling, and numerical uncertainty should be considered when applying the proposed compensation approach to actual roll-based bending processes.

## 3. Effect of Process Parameters on Springback Behavior

### 3.1. Combined Effect of Feeding Displacement and Roller Rotational Speed

In roll-based bending processes, the feeding displacement and roller rotational speed simultaneously govern the material transport and roller–material contact conditions. Rather than treating these parameters independently, their combined influence on springback behavior under practically relevant process conditions was investigated.

**Figure 4:**
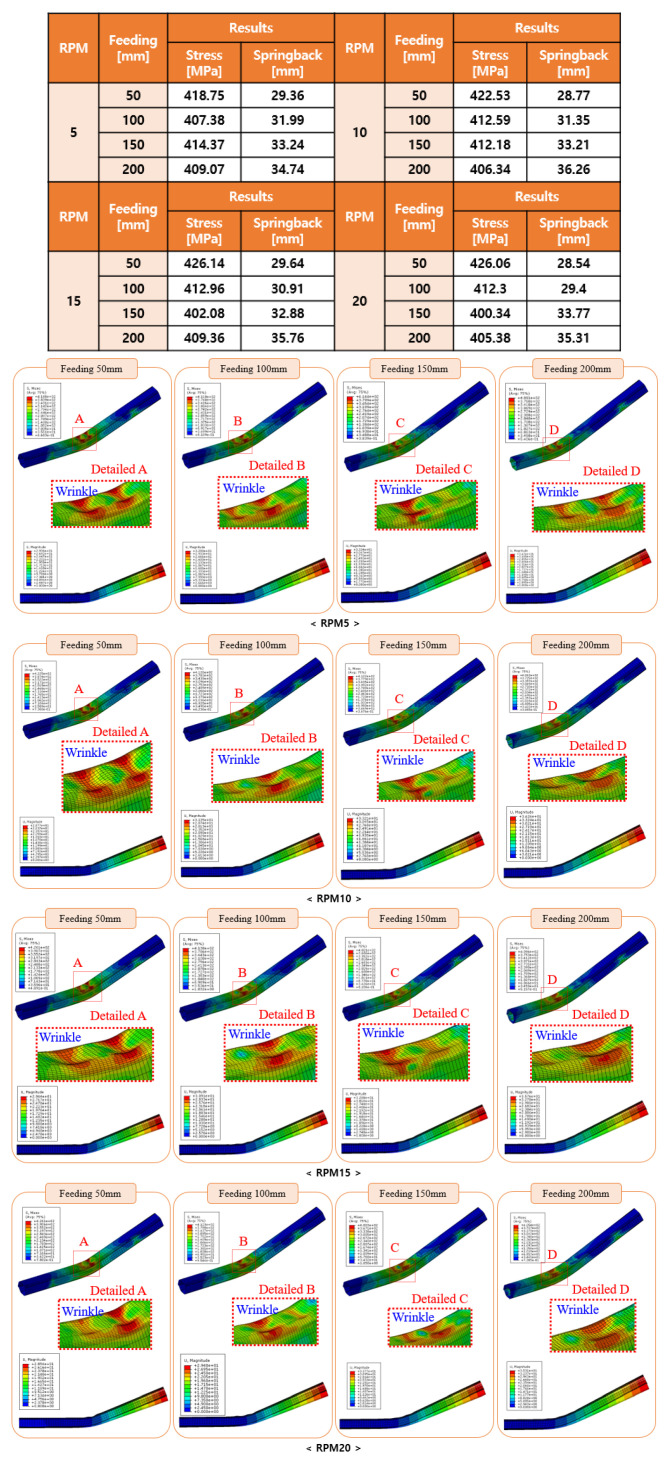
Combined effect of feeding displacement and roller rotational speed on springback behavior.

Figure 4 presents the springback behavior as a function of feeding displacement for different roller rotational speeds. The results show that the angle-based springback increased monotonically with the feeding displacement under all investigated roller speeds. This consistent trend indicates that feeding displacement directly controls the contact length and deformation history between the rollers and the extrusion, thereby acting as the primary parameter governing springback behavior. In contrast, under identical feeding displacement conditions, variations in the roller rotational speed resulted in only minor changes in the springback. Although changes in roller speed may affect local contact interactions or the material transport rate, they do not significantly alter the overall deformation history or plastic strain distribution responsible for springback. This observation is consistent with previous reports that springback is governed mainly by accumulated plastic deformation and residual stress distribution rather than deformation rate effects. Furthermore, an increase in the feeding displacement led to a noticeable rise in springback, without any substantial change in the maximum stress level. This suggests that springback behavior is influenced more strongly by the spatial distribution of deformation than by peak stress values. The same tendency was observed across all roller rotational speeds, which confirms the dominant role of feeding displacement.

Overall, these results demonstrate that springback behavior in roll-based bending is primarily controlled by the feeding displacement, while the roller rotational speed plays a secondary role, exerting limited influence under identical feeding conditions. Consequently, feeding displacement should be prioritized over roller rotational speed when designing process parameters for springback control. The limited influence of roller rotational speed can also be explained by the relatively low sensitivity of the present forming response to deformation rate and frictional heating. Under identical feeding displacement and punch stroke conditions, the final curvature, contact region, and plastic deformation zone were mainly determined by the imposed geometry rather than by the roller speed. Therefore, variations in roller speed did not substantially modify the global residual stress field after unloading. In addition, because the present simulations were conducted under non-thermal forming conditions, the effect of friction-induced heating and thermal softening was not considered dominant in the investigated speed range.

### 3.2. Effect of Mandrel Application

**Figure 5:**
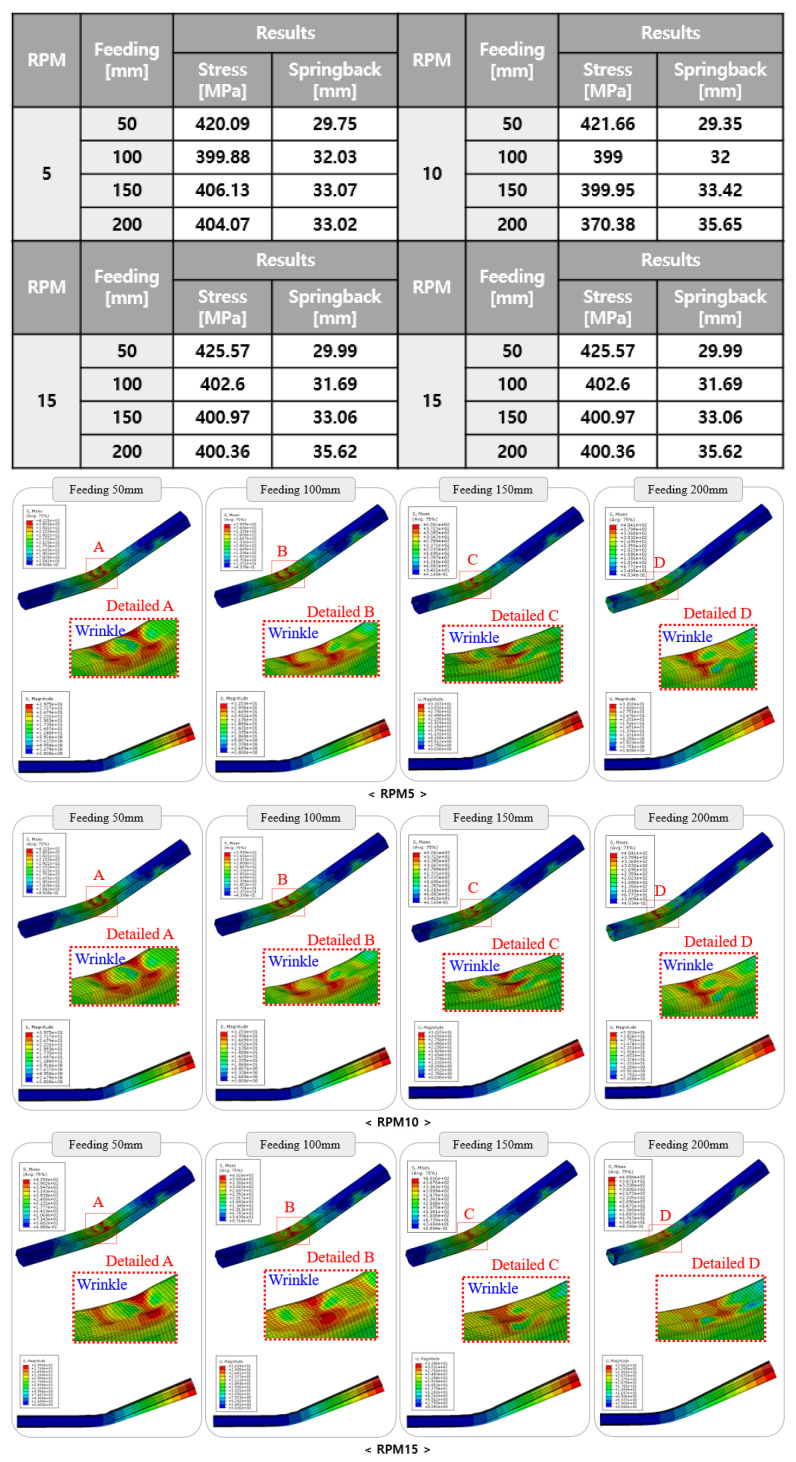

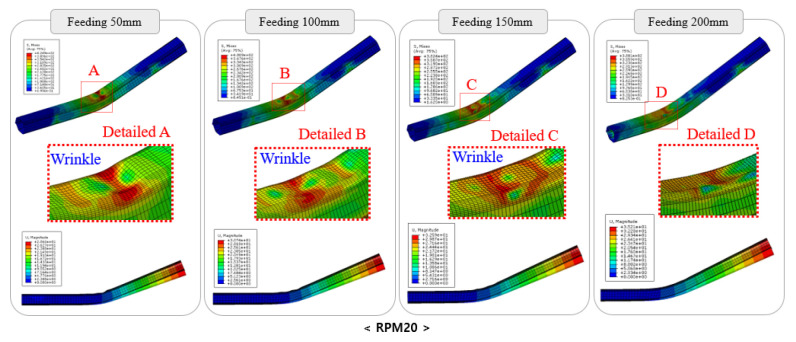
Effect of mandrel application on springback behavior.

Mandrels are commonly utilized in roll-based bending processes to provide internal support and thereby suppress cross-sectional distortion and wrinkle formation. Studies have reported that internal support conditions can influence stress distribution and forming stability, which may indirectly affect springback behavior [11,20]. The simulation results in this study indicate that the application of a mandrel generally reduces the maximum stress levels and noticeably mitigates wrinkle formation. This improvement can be attributed to the enhanced sectional stiffness provided by the mandrel, which alleviates localized deformation concentrations during bending. As shown in Figure 5, the simulation results in this study indicate that the application of a mandrel generally reduces the maximum stress levels and noticeably mitigates wrinkle formation. However, under identical feeding displacement conditions, the overall increasing trend displayed by the springback remained largely unchanged after mandrel application. This observation suggests that while mandrels are effective in improving forming stability and surface quality, they do not fundamentally alter the springback tendency, which is governed by the feeding displacement. Consequently, mandrels should be regarded as auxiliary process elements for stabilizing deformation rather than as dedicated tools for controlling springback behavior.

## 4. Stroke-Based Springback Compensation Strategy

### 4.1. Target Geometry and Definition of Springback

**Figure 6:**
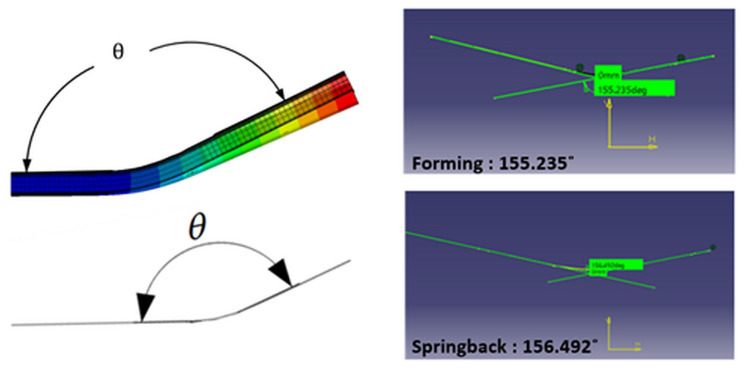
Definition of angle-based springback before and after unloading.

As defined in Section 2, the target geometry for the roll-based bending process considered in this study was a curvature radius of (R = 150) mm. The target geometry and the definitions used to quantify springback are illustrated in Figure 6. This radius was selected as an intermediate bending condition in which springback behavior could be clearly evaluated without the dominant influence of forming instabilities such as wrinkling or fracture.

In roll-based bending, springback occurs during unloading after the forming process and results in a deviation between the target geometry and the final unloaded shape. In this study, springback was evaluated using two complementary measures. Displacement-based springback was defined as the maximum deviation of the extrusion centerline between the fully loaded configuration at the end of forming and the unloaded configuration. Angle-based springback was defined as the difference between the reference bending angle corresponding to the target curvature of (R = 150) mm and the actual bending angle measured after unloading, as shown in Figure 6. These two measures allow springback behavior to be interpreted from both geometric and process-design perspectives [5,8].

### 4.2. Springback Characteristics Under Baseline Forming Condition

To obtain the target curvature of R = 150 mm, a punch roller stroke of 50 mm was employed as the baseline forming condition. Under this condition, the shape produced immediately after the forming stage was close to the target curvature. However, a pronounced springback was observed after unloading. Based on the numerical results, under the baseline stroke, the displacement-based springback reached approximately 30–40 mm. In contrast, the corresponding angle-based springback was measured to be approximately 1°. The relatively large displacement-based springback arose from the geometric amplification effect associated with the length of the extrusion, whereby a small angular change resulted in a considerable linear deviation along the longitudinal direction. Therefore, the observed displacement-based springback should not be interpreted as a local deformation comparable to the punch stroke itself but rather as the geometrically amplified outcome of a relatively small angle-based springback.

### 4.3. Compensation Mechanism Based on Stroke Adjustment

The stroke-based springback compensation strategy employed in this study is designed to introduce intentional overbending during the forming stage by increasing the punch roller stroke, which compensates for the elastic recovery during unloading. To investigate this mechanism, the punch stroke was incrementally raised from the baseline value of 50 mm, with forming and unloading simulations performed for each stroke value. The numerical results indicate that increasing the punch stroke to approximately 52.5 mm compensated for springback under the investigated finite-element conditions, yielding a final curvature that closely matched the target curvature radius of R = 150 mm after unloading. Although the stroke increment was only 2.5 mm, it induced sufficient additional overbending at the end of the forming stage. This behavior is attributed to changes in the roller–material contact length and contact geometry caused by stroke adjustment, which modified the bending moment distribution and accumulated plastic deformation history. Thus, the angle-based springback was suitably counterbalanced, and the geometric amplification of the displacement-based springback along the extrusion length was significantly suppressed after unloading. Accordingly, under the present numerical conditions, a 2.5 mm increase in the punch stroke was predicted to compensate for the displacement-based springback of approximately 30–40 mm, which is physically reasonable when considering the relationship between angular springback and geometric amplification.

**Figure 7:**
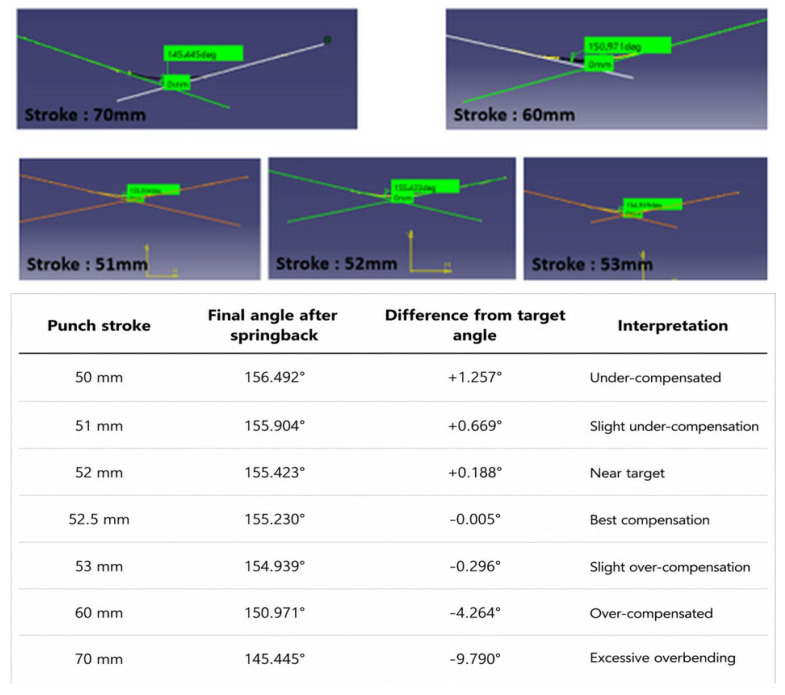
Effect of punch stroke on angle-based springback after unloading and corresponding compensation accuracy.

**Figure 8:**
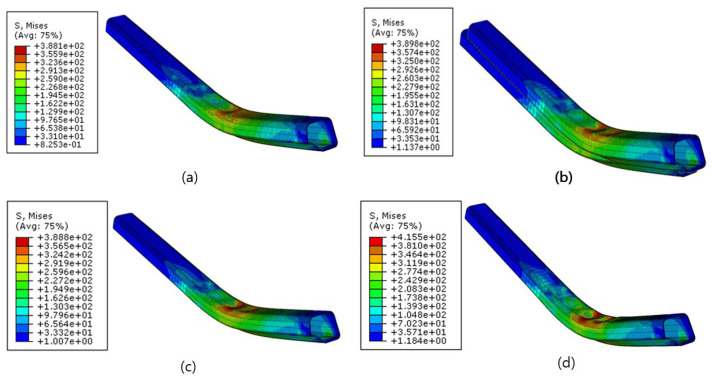
Von Mises stress distributions during forming under different punch strokes: (**a**) 50 mm, (**b**) 52 mm, (**c**) 53 mm, and (**d**) 70 mm.

To further clarify the physical mechanism of the proposed stroke-based compensation, the von Mises stress distribution during forming and the residual stress distribution after unloading were compared for different punch strokes. As shown in Figure 7 and Figure 8, the increase in punch stroke changed not only the final geometry but also the stress concentration pattern during forming and the residual stress distribution after unloading. The compensation mechanism was not merely a geometrical consequence of increasing the punch displacement; rather, it was associated with the redistribution of the stress field and the residual bending moment generated during unloading. When the punch stroke was increased from the baseline condition, the roller–extrusion contact region expanded and the plastic deformation zone near the bending center became more pronounced. This altered the stress gradient between the tensile and compressive sides of the extrusion section and consequently modified the residual stress state after unloading.

**Figure 9:**
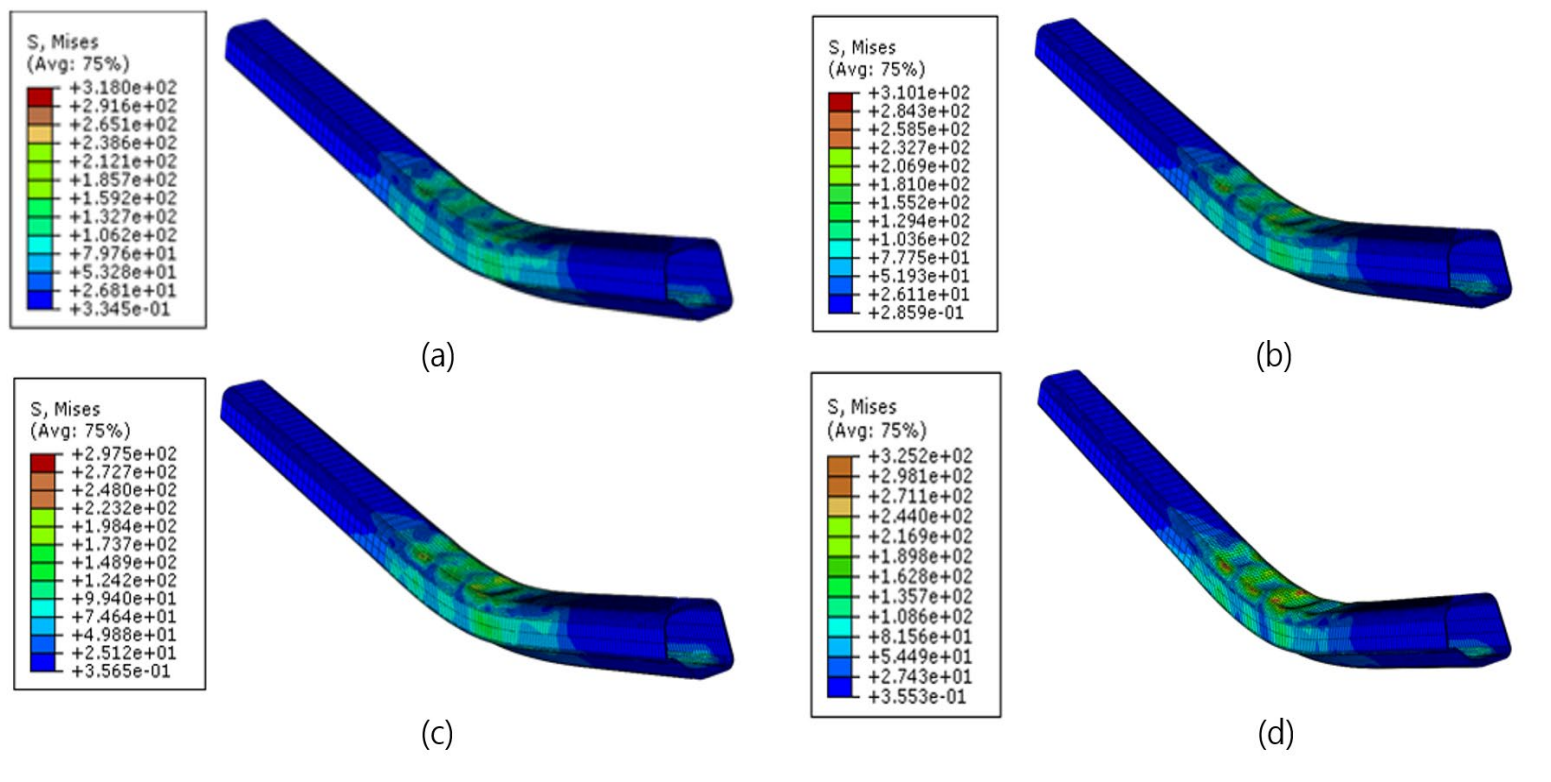
Residual stress distributions after unloading under different punch strokes: (**a**) 50 mm, (**b**) 52 mm, (**c**) 53 mm, and (**d**) 70 mm.

The final bending angles obtained after unloading for the investigated punch strokes are compared in Figure 9. Under the baseline stroke of 50 mm, the formed shape was close to the target geometry before unloading; however, the residual stress distribution generated during bending produced elastic recovery, resulting in a final angle of 156.492°. This indicates an under-compensated condition under the investigated numerical conditions. In contrast, increasing the punch stroke to 52.5 mm introduced an appropriate amount of overbending during the forming stage. After unloading, the redistribution of residual stress counterbalanced the angular springback, yielding a final angle of 155.23°, which was close to the target angle of 155.235° in the finite-element analysis. Therefore, the effective compensation stroke should be interpreted as the condition in which the residual bending moment after unloading is balanced with the intentional overbending introduced during forming.

However, when the punch stroke was further increased to 60 or 70 mm, excessive plastic deformation occurred in the bending region, and the residual stress field after unloading no longer compensated the target geometry appropriately. The final angles decreased to 150.971° and 145.445°, respectively, indicating over-compensation. These results demonstrate that stroke-based compensation has an effective process window rather than a simple monotonic improvement with increasing stroke. The optimum stroke is therefore determined by the balance between intentional overbending and residual stress-driven elastic recovery.

### 4.4. Effective Compensation Window and Implications for Process Design

A comprehensive analysis of the final curvature response as a function of punch stroke revealed the existence of a stroke compensation window within which the unloaded curvature remained close to the target curvature radius of R = 150 mm under the investigated finite-element conditions. Within this range, the relationship between stroke adjustment and final curvature response exhibited a relatively monotonic tendency, suggesting that an appropriate compensation stroke can be numerically estimated during process design.

Compared with conventional springback compensation techniques based on iterative finite-element simulations or die-shape modification [5,9], the proposed simulation-based stroke compensation approach may offer practical advantages because it relies on the adjustment of controllable process parameters rather than structural modification of the forming tools. However, because the present study was conducted entirely using finite-element simulations, the proposed compensation window should be interpreted as a numerical guideline rather than as a fully validated industrial process condition.

Together with the feeding displacement-dominant behavior described in Section 3, the numerical results suggest that springback control in roll-based bending may be achieved through coordinated control of feeding displacement and punch stroke under appropriately selected process conditions. Therefore, the proposed approach provides a numerical basis for developing practical process guidelines to improve geometric accuracy in roll-based bending processes for high-strength aluminum extrusions. Further experimental validation is required to confirm the effectiveness of the proposed compensation approach in actual roll-bending processes.

## 5. Conclusions

In this study, the springback behavior of high-strength aluminum extrusions subjected to roll-based bending was investigated through finite-element analysis, and a simulation-based stroke compensation approach was proposed. The effects of feeding displacement, roller rotational speed, and internal mandrel application were comparatively evaluated.

The results showed that feeding displacement was the dominant process parameter governing springback behavior, whereas roller rotational speed had only a limited influence under identical feeding conditions. Internal mandrel application reduced stress concentration and suppressed wrinkle formation, thereby improving forming stability; however, it did not fundamentally change the overall springback tendency.

For the target curvature radius of R = 150 mm, the baseline punch stroke produced springback after unloading, while an increased punch stroke numerically compensated for the angle-based springback under the investigated finite-element conditions. The compensation mechanism was associated with intentional overbending, changes in roller–material contact geometry, and residual stress redistribution during unloading. These results suggest that coordinated control of feeding displacement and punch stroke may support springback reduction in roll-based bending processes.

Overall, the proposed simulation-based stroke compensation approach provides a numerical basis for developing practical process guidelines for high-strength aluminum extrusion bending. Since the present study was based entirely on finite-element simulations, the proposed approach should be regarded as a preliminary numerical guideline. Experimental validation and further investigation of material anisotropy, the Bauschinger effect, friction conditions, mesh convergence, and time-step sensitivity are required before direct application to industrial roll-based bending processes.

## Figures and Tables

**Table 1:** Material properties and contact parameters used in the finite-element simulation.

Category	Parameter	Value	Unit
Elastic property	Young’s modulus	70,000	MPa
	Poisson’s ratio	0.33	-
	Density	2.70 × 10^−9^	tonne/mm^3^
Plastic property	Plastic behavior	True stress-true strain curve	-
	Hardening model	Isotropic hardening	-
Contact condition	Contact formulation	Surface-to-surface contact	-
	Friction model	Coulomb friction	-
	Friction coefficient	0.12	-

## Data Availability

The original contributions presented in this study are included in the article. Further inquiries can be directed to the corresponding author.

## Abbreviations

θt	Target bending angle before springback (°)
θs	Bending angle after springback (°)
δs	Springback displacement after unloading (mm)
δf	Feeding displacement (mm)
δp	Punch roller stroke (mm)

## References

[1] Miller W.S., Zhuang L., Bottema J. (2000). Recent development in aluminium alloys for the automotive industry. Mater. Sci. Eng. A.

[2] Hirsch J. (2014). Recent development in aluminium for automotive applications. Trans. Nonferrous Met. Soc. China.

[3] Wagoner R.H., Lim H., Lee M.G. (2013). Advanced issues in springback. Int. J. Plast..

[4] Livatyali H., Altan T. (2001). Prediction and elimination of springback in straight flanging using computer-aided design methods: Part 1. Experimental investigations. J. Mater. Process. Technol..

[5] Gan W., Wagoner R.H. (2004). Die design method for sheet springback. Int. J. Mech. Sci..

[6] Zhao H., Zhang G., Yin Z., Wu L. (2012). Three-dimensional finite element analysis of thermal stress in single-pass multi-layer weld-based rapid prototyping. J. Mater. Process. Technol..

[7] Zavadskas E., Kalibatas D., Kalibatiene D. (2016). A multi-attribute assessment using WASPAS for choosing an optimal indoor environment. Arch. Civ. Mech. Eng..

[8] Lingbeek R., Meinders T., Huétink J. Springback Compensation: Fundamental Topics and Practical Application. Proceedings of the ESAFORM 2006 Conference.

[9] Carpinteri A., Paggi M., Zavarise G. (2008). The effect of contact on the decohesion of laminated beams with multiple microcracks. Int. J. Solids Struct..

[10] Krell J., Röttger A., Geenen K., Theisen W. (2018). General investigations on processing tool steel X40CrMoV5-1 with selective laser melting. J. Mech. Work. Technol..

[11] Scholz-Reiter B., Kück M., Lappe D. (2014). Prediction of customer demands for production planning—Automated selection and configuration of suitable prediction methods. CIRP Ann..

[12] Wang A., Zhong K., El Fakir O., Liu J., Sun C., Wang L.-L., Lin J., Dean T.A. (2020). Analysis and control of twist defects of aluminum profiles with large Z-section in roll bending process. Metals.

[13] Chen P., Lu S. (2024). Springback characteristics and influencing laws of four-axis flexible roll bending forming for aluminum alloy. PLoS ONE.

[14] Moreira M.d.S., Guilherme C.E.M., de Souza J.H.C., dos Santos E.D., Isoldi L.A. (2025). Numerical Analysis of the Three-Roll Bending Process of 6061-T6 Aluminum Profiles with Multiple Bending Radii Using the Finite Element Method. Metals.

[15] Cao H., Yu G., Liu T., Fu P., Huang G., Zhao J. (2023). Research on the curvature prediction method of profile roll bending based on machine learning. Metals.

[16] Gao E., Xue D., Li Y. (2025). Springback angle prediction for high-strength aluminum alloy bending via multi-stage regression. Metals.

[17] Zhai R., Ding X., Yu S., Wang C. (2018). Stretch bending and springback of profile in the loading method of prebending and tension. Int. J. Mech. Sci..

[18] Hou R., Wang T., Lv Z., Tian Y. (2018). Investigation of the pulsed waterjet flow field inside and outside of the nozzle excited by ultrasonic vibration. Int. J. Adv. Manuf. Technol..

[19] Karafillis A.P., Boyce M.C. (1992). Tooling design in sheet metal forming using springback calculations. Int. J. Mech. Sci..

[20] Bong H.J., Lee J., Lee M.G. (2025). Finite Element Modeling of Springback Behavior for Aluminum 6000 Series Sheets Using Three-Point Bending Tests. Int. J. Automot. Technol..

[21] Saleem K.B., Koufi L., Alshara A.K., Kolsi L. (2020). Double-diffusive natural convection in a solar distiller with external fluid stream cooling. Int. J. Mech. Sci..

[22] Delannay L., Melchior M., Signorelli J., Remacle J.-F., Kuwabara T. (2009). Influence of grain shape on the planar anisotropy of rolled steel sheets—Evaluation of three models. Comput. Mater. Sci..

[23] Barlat F., Aretz H., Yoon J.W., Karabin M.E., Brem J.C., Dick R.E. (2005). Linear transformation-based anisotropic yield functions. Int. J. Plast..

